# Pathological Fractures in Patients Affected by Pycnodysostosis: A Case Series

**DOI:** 10.3390/jcm13092522

**Published:** 2024-04-25

**Authors:** Maria Beatrice Bocchi, Cristina Giuli, Francesco Farine, Camilla Ravaioli, Sara Martellini, Pasquale Farsetti, Osvaldo Palmacci

**Affiliations:** 1 Department of Orthopaedics, Fondazione Policlinico Universitario A. Gemelli IRCCS, 00168 Rome, Italy; mariabeatrice.bocchi01@icatt.it (M.B.B.); francesco.farine@gmail.com (F.F.); martellini0897@gmail.com (S.M.); osvaldo.palmacci@policlinicogemelli.it (O.P.); 2 Scuola di Specializzazione in Ortopedia e Traumatologia, Università Cattolica del Sacro Cuore, 00168 Rome, Italy; 3 Department of Clinical Science and Translational Medicine, Section of Orthopaedics and Traumatology, University of Rome Tor Vergata, 00133 Rome, Italy

**Keywords:** pycnodysostosis, rare disease, pathological fractures, nonunion, refracture

## Abstract

**Background/Objectives**: Pycnodysostosis is a rare genetic disorder causing skeletal dysplasia. It is determined by a gene mutation leading to cathepsin K deficiency and predisposes a patient to osteosclerosis, resulting in increased bone fragility. The altered bone quality typical of this disease is responsible for an increased risk of fractures. The purpose of our study was to evaluate the orthopedic manifestations and potential pitfalls in the surgical treatments of pathological fractures in a series of patients treated in our institution who were affected by pycnodysostosis. **Methods**: We retrospectively evaluated clinical and radiographic characteristics of five patients with pycnodysostosis treated for pathological fractures at our hospital in the past 5 years. **Results**: Two male and three female patients were included in this study. Four patients had a family history of pycnodysostosis. All the patients were of short stature, but only two underwent growth hormone treatment. All the patients experienced fractures, mostly in their lower limbs and occurring as a result of low-energy trauma. Most of the patients experienced either consolidation delay or nonunion. **Conclusions**: The orthopedic management of fractures in patients with pycnodysostosis poses an ongoing challenge for orthopedic surgeons. The fact that the bone is simultaneously sclerotic and brittle makes any orthopedic surgical treatment challenging and at a high risk of nonunion in any case.

## 1. Introduction

Pycnodysostosis (PYCD) is a rare autosomal recessive lysosomal storage disease of the bone [1]. The prevalence of the disease is estimated to be 1 to 1.7 per million and is the highest in consanguineous populations, with equal sex distribution [2]. Less than 500 affected individuals have been reported in the medical literature [3].

Individuals affected by this rare condition have distinctive characteristics, including short stature, osteosclerosis of the long bones and resulting recurrent fractures, specific facies features (including a prominent forehead, maxillary hypoplasia, a small chin, and an obtuse mandibular angle), delayed closure of cranial sutures, and acro-osteolysis of the distal phalanx with dystrophic nails and short fingers [3,4,5]. The genotypic basis of PYCD is derived from a loss-of-function mutation of the cathepsin K gene (CTSK) located on chromosome 1q21 [6]. Cathepsin K is a lysosomal cysteine protease highly expressed in osteoclasts, where it is essential for the degradation of collagen type 1, which constitutes 95% of organic bone matrix. Due to the loss-of-function mutations, patients with pycnodysostosis have a normal number of osteoclasts, but these are functionally unable to adequately degrade the organic matrix, leading to increased bone density [4,7,8,9]. Because of the altered bone, a significant manifestation of PYCD is a high susceptibility to spontaneous or low-trauma fractures and a significant delay in fracture healing, which poses a considerable challenge for orthopedic treatments [10]. Individuals with PYCD have an increased fracture rate, with an average of 0.2 fractures per year and delayed healing with incomplete remodeling [11]. From data extrapolated in the literature review conducted on the topic, Taka et al. [12] inferred that 88.1% of the population affected by PYCD had a history of fracture, of which 44.2% were reported as spontaneous or caused by minor trauma.

Surgical fixation is quite challenging and is often complicated by narrow medullary canals and sclerotic and brittle bone, with an increased risk of intraoperative iatrogenic fractures [13]. For these reasons, to date, it is still debated whether the preferred treatment for these patients in case of fractures is either primary, closely approximating the fracture edges with a rigid fixation as in plate fixation, or secondary bone healing, bringing the ends close enough together while still leaving some residual movement as in intramedullary synthesis [14]. However, again, because of the altered bone quality typical of this condition, delayed union also remains a problem [15].

Here, we report a series of pathological fractures and their treatments in patients with pycnodysostosis, which have been followed entirely, or at least partially, at our institution.

## 2. Materials and Methods

This retrospective study included a series of five patients attending the Paediatric Orthopaedic Service of our hospital from November 2020 to December 2023 who presented with a pycnodysostosis diagnosis.

We recorded gender, age at presentation at our orthopedic pediatric service, family history of pycnodysostosis, genetic diagnosis, number of fractures that had occurred, treatment received, and possible complications. This study respects national ethical standards and the Declaration of Helsinki. Written informed consent for surgical and clinical data collection for scientific purposes was obtained from all patients/parents or guardians at admission and before surgery, according to institutional protocol.

## 3. Results

Our series included five patients, and their general profiles are summarized in Table 1.

### 3.1. Case Descriprion

#### 3.1.1. Case 1

A 53-year-old woman, born from consanguineous Caucasian parents, was diagnosed with PYCD at 6 months of age in a different hospital. She had a younger brother (Case 2) affected by the same disorder and a positive family history of other PYCD cases. She presented with typical cranio-facial dimorphisms and increased bone density and underwent several maxillo-facial surgeries, including mandibulectomy, during her childhood. Hand radiographs showed partial dysplasia of the distal phalanx, clinically evident as malformed and dysmorphic fingers. She had been diagnosed with short stature, but she did not receive pharmacological therapy with growth hormones. She did not manifest other systemic comorbidities, but she experienced multiple fractures over the years as a result of low-energy trauma. At the age of 13 years, she experienced a tibial and fibular shaft fracture treated surgically by closed reduction and internal fixation with an intramedullary nail at a different hospital. At the 6-month follow-up, she was diagnosed with pseudarthrosis and received an autologous bone graft from her homolateral iliac crest. Her fracture healed completely, and she was able to return to her normal daily life activities in almost one year. At the age of 47 years, she was diagnosed with a proximal femur fracture, treated with open reduction and internal fixation with a Compression Hip Screw system (CHS) plate. The fracture healed uneventfully in six months with a regular post operative follow-up. Six months later, a proximal femur fracture of the contralateral side occurred and was treated by open reduction and internal fixation with the same surgical device and technique. The fracture healed uneventfully in 6 months with regular post operative follow-up. At the age of 53, the patient came to the emergency room of our hospital and then to the attention of our Service because she refractured her left femur at the distal end of the CHS plate previously placed. The patient was treated by removing the previous plate, and then a new open reduction and osteosynthesis procedure with a Dynamic Hip Screw (DHS) plate was performed. The fracture healed completely in 6 months. To date, the patient has returned to her normal daily life activities.

#### 3.1.2. Case 2

A 37-year-old man born to consanguineous Caucasian parents with a family history of PYCD was diagnosed with PYCD. His older sister (Case 1) was affected by the same rare disease. He was also diagnosed with Down Syndrome. His anamnestic history was characterized by severe sleep apnea syndrome, bilateral tympanosclerosis, short stature, typical cranio-facial dimorphisms, and mid-face retrusion; he had undergone Le Fort III advancement osteotomy in his childhood. At the age of 30, the patient had cardiopulmonary arrest during hospitalization for pneumonia that led him to be tracheostomized. At the age of 37 years, low-energy trauma resulted in a subtrochanteric fracture, which was treated with open reduction and internal fixation with a DHS plate in a different hospital. Two months later, the patient was hospitalized for acute respiratory distress in our clinic. During the stay, a spontaneous femoral refracture occurred. The patient was then transferred to the orthopaedic department and treated with closed reduction and intramedullary osteosynthesis with an Adolescent Lateral Femoral Nail (diameter 8.2 mm) due to the shortness and narrowness of the femur. Due to the high level of bone stiffness, the surgery was complicated by a iatrogenic fracture, and therefore it was necessary to complete the osteosynthesis with a cerclage. Post-operative management included early range-of-motion without weightbearing for the first 6 weeks, and then with progressive weightbearing gradually introduced according to his comorbidities. At the age of 39, the patient returned to our attention because of new onset anterolateral thigh pain. A TC scan showed complete healing of the proximal femur fracture and pseudoarthrosis at the level of the previous iatrogenic fracture of the femoral diaphysis. In view of the patient’s poor clinical condition, in accordance with the family, the decision was made to proceed with conservative treatment. To date, the patient uses a wheelchair.

#### 3.1.3. Case 3

A 16-year-old girl was followed at the Pediatric Rare Diseases Department of our hospital since birth. She was born to non-consanguineous Caucasian parents with a younger brother (Case 4) affected by the same illness and no family history of PYCD. According to genetic testing, the patient had homozygous mutation c.721CT of the CTSK gene. Her anamnestic history was characterized by mild–moderate OSAS, pharmacologically treated hypothyroidism, typical cranio-facial dimorphisms with megacephaly, malformed and dysmorphic fingers, papilledema, eye disorders such as hypermetropia and astigmatism, bilateral flat feet, and L5-S1 anterolisthesis. At the age of 13, she started growth hormone treatment because of her short stature, which was discontinued after three years because of the onset of secondary amenorrhea and significant weight loss. The patient experienced multiple fractures because of low-energy trauma over the years. At the age of 10, she experienced a non-displaced middle-third clavicle fracture, which was treated non operatively with a brace as per indication of the emergency room orthopedist. The fracture healed uneventfully in 3 months. At the age of 16, she experienced a compound IV–V metatarsal fracture, which was treated conservatively by cast immobilization, and an L4 compression fracture, which was treated with a corset. At the age of 17, she fractured her right tibia and fibula. The fracture was displaced and was therefore treated by open reduction and internal fixation with a seven-hole plate and screws. The fracture healed uneventfully in 3 months. To date, the patient has returned to her normal daily life activities.

#### 3.1.4. Case 4

A 14 -year-old boy was followed at the Pediatric Rare Diseases Department of our hospital since birth. He was born to two non-consanguineous Caucasian parents. He had an older sister (Case 3) with the same illness and no family history of PYCD. According to genetic testing, the patient had homozygous mutation c.721CT of the CTSK gene. His anamnestic history was characterized by severe sleep apnea, cranio-facial dimorphisms with megacephaly, malformed and dysmorphic fingers, papilledema, impacted and mispositioned teeth, and genu and ankle valgus. At the age of 9 years, he started growth hormone treatment because of his short stature, which was discontinued because of the onset of bilateral hip epiphysiolysis at the age of 14. This bilateral hip epiphysiolysis was treated surgically trough bilateral pinning with cannulated screws. Approximately one year after surgery, the patient again took up growth hormone treatment, which is currently ongoing. At the age of 15, he experienced a non-displaced metatarsal fracture, which was treated non-operatively with cast immobilization for 4 weeks. The fracture healed in 2 months.

#### 3.1.5. Case 5

A 28-year-old woman born to consanguineous Caucasian parents and with no family history of PYCD was diagnosed with PYCD. Her anamnestic history was characterized by thrombocythemia due to JAK 2 mutation, typical cranio-facial dimorphism with megacephaly, crowded teeth, malformed and dimorphic fingers on both hands and feet, and short stature, for which she had never taken medical treatment. Over the years, she had suffered several bilateral femoral and tibial fractures, which were treated non-operatively, with complete healing occurring approximately 3 months after. All the fractures were caused by low-energy trauma. At the age of 25, she experienced a displaced left-shaft femoral fracture, which was treated in another hospital by closed reduction and internal fixation with one intramedullary nail. The fracture never healed and evolved into pseudoarthrosis. At the age of 28, after a low-energy trauma event, she experienced a refracture on the exact same location of the previous one with nail breakage. She was treated surgically by removing the nail and by performing a new closed reduction and synthesis with elastic intramedullary stable nailing (ESIN). To date, the patient walks with sporadic thigh pain and complete weightbearing but not without a crutch. However, the fracture has again resulted in pseudoarthrosis; therefore, a new surgery will be necessary.

### 3.2. Demographic Features

Three patients were female, and two were male. The average age at the time of admission at our institution was 26.9 (14–53) years old. Owing to the autosomal recessive nature of the disease, 2/5 cases reported family history of PYCD, and 3/5 had consanguineous parents. Among the patients in our series, 4/5 were siblings (two pairs).

Only 2/5 patients of our series, who were siblings followed at our hospital’s Pediatric Rare Diseases Department, were known to have a specific mutation—the homozygous mutation c.721CT of the CTSK gene. In the remaining cases, the genetic records could not be traced.

### 3.3. Patient’s Clinical Characteristics

All the patients (5/5) were of short stature, for which 2/5 patients underwent GH treatment, which was discontinued after three years in both cases because of the onset of secondary amenorrhea and bilateral hip epiphysiolysis, respectively. Case 4 resumed the drug after about a year and is currently undergoing treatment. Further orthopedic characteristics found in our population include genu valgus (Case 4), ankle valgus (Case 4), flat feet (Case 2 and Case 3), and L5-S1 spondylolysis with spondylolisthesis (Case 3).

### 3.4. Pathological Fractures and Surgical Management

All the patients in the series (5/5) reported a history of fractures that occurred spontaneously or as a result of low-energy trauma. The total number of fractures recorded was 17. Among them, 4/17 were tibial shaft fractures, 5/17 were proximal femur fractures (of which 2 were hip epiphysiolysis and 3 were subtrochanteric fractures), 3/17 were femur shaft fractures, 3/17 were metatarsal fractures, 1/17 was a clavicle fracture, and 1/17 was a spinal fracture.

Of the treated PYCD patients, 8/17 were treated with surgical management, while 9/17 were treated conservatively. Of the 8 fractures surgically treated, the majority (4/8) were treated with internal plate fixation, while 2/8 were treated with intramedullary fixation. The remaining fractures were treated with other surgical methods such as screw fixation (2/8).

### 3.5. Complications

Three patients (3/5) experienced refracture, and the overall refracture rate was 18% (3/17). The refracture rate was higher in the internal plate fixation (2/3) versus the intramedullary fixation (1/3).

A refracture of the femoral shaft distal to the plate previously placed occurred in Case 1 because of a low-energy trauma. It was managed at our institution by removing the previous plate and performing an open reduction, as well as a new osteosynthesis with a longer plate and screws. Case 2 also experienced refracture of the femoral diaphysis previously treated with a plate. In this occasion, it turned out to be a spontaneous fracture that was treated in our center with closed reduction and an osteosynthesis with an intramedullary long nail. Intra-operatively, the surgery was complicated, with a femoral shaft fracture distal to the former that was treated with cerclages. Finally, even Case 5 experienced a diaphyseal femoral refracture as a result of a low-energy trauma, which was initially treated with an intramedullary device and developed into pseudoarthrosis. The fracture was treated with device removal and new osteosynthesis with two 4 mm titanium elastic nails.

We observed four cases of delayed consolidation (23.5%) and four cases of pseudoarthrosis (23.5%) (Table 2). Among the latter, in one case, the patient successfully received an autologous bone graft from her homolateral iliac crest (Case 1); in Case 2, a decision was made not to further intervene in view of the patient’s general poor clinical conditions. The last two cases of pseudarthrosis occurred at the same femoral diaphyseal fracture in Case 5. Among all the surgeries, also considering the treatment of refractures and pseudarthrosis, 6/11 were performed at our institution.

## 4. Discussion

Due to their genetically determined, altered bone quality, patients affected by Pycnodysostosis have an increased susceptibility to pathological fractures.

The bones of the patients affected by this disease are both dense and brittle, such that minimal trauma can easily lead to fractures [16]. In our experience, all cases of reported fractures were the result of low-energy trauma or even spontaneous fractures.

To date, there is no unanimity about the optimal treatment of fractures in patients with PYCD, as indicated in the most recent systematic reviews of the literature on the topic [10,12].

**Figure 1:**
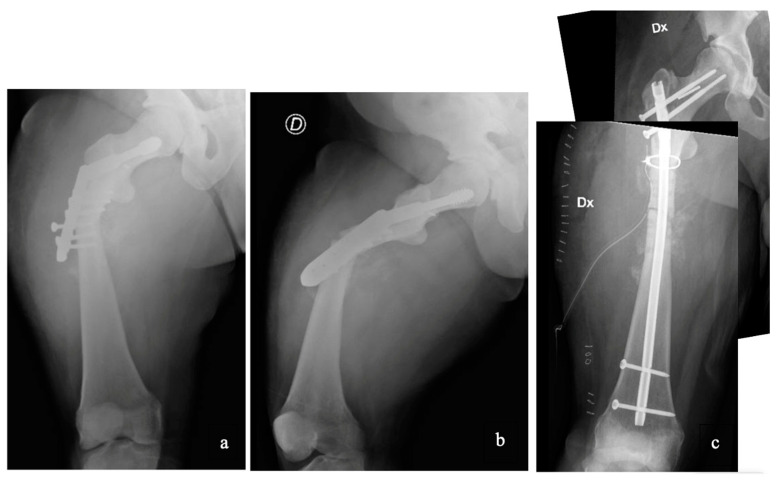
Case 2 had a subtrochanteric refracture (**a**,**b**). During canal reaming, the surgery was complicated by an intraoperative fracture that we managed with a cerclage application before proceeding with the long nail placement (**c**).

As already specified in the results, not all of the surgeries we referred to were performed at our hospital. However, as much as our experience in treating patients with this condition is relative, by comparing it with what has been reported in the literature, we found many of the difficulties already described [17,18,19,20,21,22]. Intramedullary synthesis in these patients is extremely difficult because of the narrowness of the canal, but also because of the sclerotic nature of the bone, which makes reaming extremely difficult and risky [23,24,25]. In Case 2, we decided to treat the subtrochanteric refracture with previous synthesis removal and intramedullary nail synthesis. However, during canal reaming with progressive burs, the surgery was complicated by an intraoperative fracture that we managed with a cerclage application before proceeding with the long nail placement (Figure 1). In addition, what we found months later during outpatient follow-up was the healing of the refracture foci and, conversely, the nonunion of the iatrogenic fracture. In this specific case, considering the generally poor clinical conditions, we decided not to surgically intervene further.

**Figure 2:**
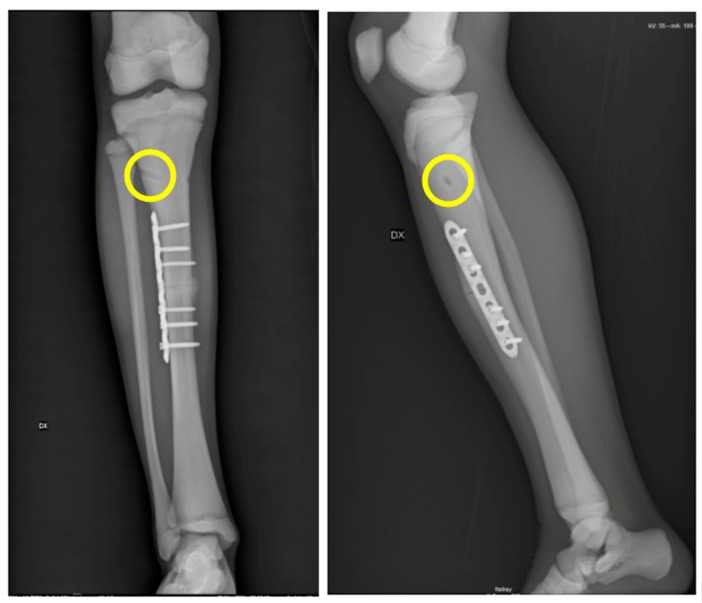
Case 3 had a displaced tibia shaft fracture that we initially planned to synthesize with two elastic titanium nails. However, during surgery, we experienced several difficulties throughout the preparation of the entrance of the elastic nail (circle) and then also in an attempt to advance the nail along the canal. Therefore, we proceeded with an open reduction and plate fixation.

Case 3 had a displaced tibia shaft fracture as a consequence of a low-energy trauma that occurred during pediatric age. We initially planned to synthesize the fracture with elastic intramedullary stable nailing (ESIN), as it was deemed a less invasive surgery in view of the patient’s age. However, during surgery, we experienced several difficulties from the beginning. These occurred first throughout the preparation of the entrance of the elastic nail due to the extreme thickness of the cortical and, second, in an attempt to advance the nail along the basically inexistent canal. Therefore, we found ourselves forced to change the ongoing approach by then proceeding with an open reduction and plate fixation. Although we completed the surgery with a satisfying result, we faced a few difficulties. During the drilling, in fact, the feeling of the bone was as if managing marble, to the extent that we risked drill breakage on a number of occasions (Figure 2). Fortunately, the fracture healed on time, and there were no complications at the follow up.

**Figure 3:**
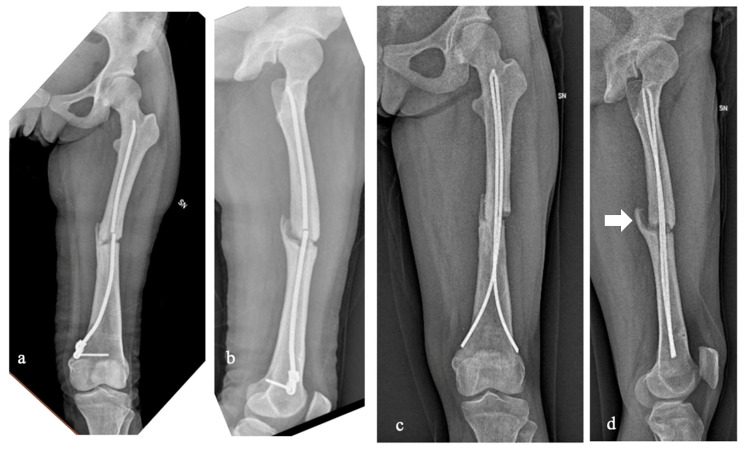
Case 5 had a displaced diaphyseal left femur fracture that never healed and, as a result of a low-energy traumatic event, the femur refractured at the pseudoarthrosis site with associated nail breakage (**a**,**b**). We decided to treat the refracture with two 4 mm titanium elastic nails placed with the standard technique (ESIN, elastic stable intramedullary nailing). Within a year from the surgery, the X-rays showed a hypertrophic pseudoarthrosis (arrow) that will require a new surgery (**c**,**d**).

The intrinsically altered clastic functions affect physiological bone healing processes [20,26,27]. In our experience, the rate of delayed union and nonunion were both fairly high. Case 5 had a displaced diaphyseal left femur fracture that was intramedullary treated with a single elastic nail at a different hospital (Figure 3). The fracture never healed, and, as a result of a low-energy traumatic event, the femur refractured at the pseudoarthrosis site with associated internal device breakage. The patient then came to our attention, and we decided to treat the refracture again with an intramedullary device in order to be minimally invasive. To prevent reaming complications and due to the small size of the femur, we decided to use two 4 mm titanium elastic nails placed with the ESIN standard technique. Within a year from the surgery, despite the patient’s overall good recovery for common daily life activities, X-rays showed a hypertrophic pseudoarthrosis that will require a new surgery. Therefore, despite the fact that the stature and the weight of the patient were compatible with the ESIN technique, the patient’s age, the underlying bone disease, and the previous history of pseudoarthrosis can be considered consistent unfavorable elements. We have no direct experience of treating pseudoarthrosis with autologous bone grafts in PYCD. However, our indirect experience (Case 1) is positive, such that we could consider it a viable option for additional surgical time in Case 5.

## 5. Conclusions

In conclusion, the treatment of fractures in patients affected by PYCD remains difficult in technical terms and, as far as the intervention is planned, not infrequently unpredictable due to the altered bone quality and variable conformation. Furthermore, as much as all these patients share the same genetically determined osteoclastic disfunction, it is difficult to be able to always use the same orthopedic approach since so much variability exists within the same pathology. Therefore, it is important to evaluate each case individually, planning the most appropriate surgery given the type of fracture, the patient’s history, and the radiographic appearance while remaining prepared to modify the initial plan.

## Figures and Tables

**Table 1:** Patients’ profiles (CTSK, cathepsin K gene).

	Siblings		Siblings		
	Case 1	Case 2	Case 3	Case 4	Case 5
Sex	F	M	F	M	F
Age (years)	53	37	16	14	28
Family history	yes	yes	no	no	no
Consanguineous parents	yes	yes	no	no	yes
CTSK mutation	-	-	c.721CT	c.721CT	-
Other conditions	-	Down Syndrome	-	-	-
Short stature	yes	yes	yes	yes	yes
GH treatment	no	no	yes	yes	no
Total number of fractures	3	1	5	3	5
Fracture site:					
Proximal femur	2/3	1/1	-	2/3	-
Femur shaft	-	-	-	-	3/5
Tibial shaft	1/3	-	1/5	-	2/5
Clavicle	-	-	1/5	-	-
Metatarsal	-	-	2/5	1/3	-
Spinal	-	-	1/5	-	-
Conservative treatement	0/3	0/1	4/5	1/3	4/5
Surgical treatement	3/3	1/1	1/5	2/3	1/5
Nailing	x				x
Plate fixation	xx	x	x		
Other				xx	
Refracture	1	1	-	-	1
Delayed consolidation	2	-	-	-	2
Pseudoarthrosis	1	1	-	-	2

**Table 2:** Surgical management of fractures and outcomes.

Treatment Approach (%)		
Surgical	47%	
Conservative	53%	
Rate of specific surgical treatment approach and refracture rate	Surgical treatment (%)	Refracture (%)
Internal plate fixation	50%	67%
Intramedullary nail fixation	25%	33%
Other	25%	-
Complications (%)		
Refracture	18%	
Delayed union	23.5%	
Pseudoarthrosis	23.5%	

## Data Availability

Data are contained within the article.
